# Evaluation of the N_2_O Rate of Change to Understand the Stratospheric Brewer‐Dobson Circulation in a Chemistry‐Climate Model

**DOI:** 10.1029/2021JD036390

**Published:** 2022-11-13

**Authors:** Daniele Minganti, Simon Chabrillat, Quentin Errera, Maxime Prignon, Douglas E. Kinnison, Rolando R. Garcia, Marta Abalos, Justin Alsing, Matthias Schneider, Dan Smale, Nicholas Jones, Emmanuel Mahieu

**Affiliations:** ^1^ Royal Belgian Institute for Space Aeronomy BIRA‐IASB Brussels Belgium; ^2^ Institute of Astrophysics and Geophysics UR SPHERES University of Liège Liège Belgium; ^3^ Now at: Department of Earth, Space and Environment Chalmers University of Technology Gothenburg Sweden; ^4^ National Center for Atmospheric Research Boulder CO USA; ^5^ Universidad Complutense de Madrid Madrid Spain; ^6^ Oskar Klein Centre for Cosmoparticle Physics Department of Physics Stockholm University Stockholm Sweden; ^7^ Imperial Centre for Inference and Cosmology Department of Physics Imperial College London Blackett Laboratory London UK; ^8^ Institute of Meteorology and Climate Research (IMK‐ASF) Karlsruhe Institute of Technology Karlsruhe Germany; ^9^ National Institute of Water and Atmospheric Research Lauder New Zealand; ^10^ School of Chemistry University of Wollongong Wollongong Australia

**Keywords:** stratospheric circulation, Brewer‐Dobson circulation, tracer transport, trends, nitrous oxide

## Abstract

The Brewer‐Dobson Circulation (BDC) determines the distribution of long‐lived tracers in the stratosphere; therefore, their changes can be used to diagnose changes in the BDC. We evaluate decadal (2005–2018) trends of nitrous oxide (N_2_O) in two versions of the Whole Atmosphere Chemistry‐Climate Model (WACCM) by comparing them with measurements from four Fourier transform infrared (FTIR) ground‐based instruments, the Atmospheric Chemistry Experiment Fourier Transform Spectrometer (ACE‐FTS), and with a chemistry‐transport model (CTM) driven by four different reanalyses. The limited sensitivity of the FTIR instruments can hide negative N_2_O trends in the mid‐stratosphere because of the large increase in the lowermost stratosphere. When applying ACE‐FTS measurement sampling on model datasets, the reanalyses from the European Center for Medium Range Weather Forecast (ECMWF) compare best with ACE‐FTS, but the N_2_O trends are consistently exaggerated. The N_2_O trends obtained with WACCM disagree with those obtained from ACE‐FTS, but the new WACCM version performs better than the previous above the Southern Hemisphere in the stratosphere. Model sensitivity tests show that the decadal N_2_O trends reflect changes in the stratospheric transport. We further investigate the N_2_O Transformed Eulerian Mean (TEM) budget in WACCM and in the CTM simulation driven by the latest ECMWF reanalysis. The TEM analysis shows that enhanced advection affects the stratospheric N_2_O trends in the Tropics. While no ideal observational dataset currently exists, this model study of N_2_O trends still provides new insights about the BDC and its changes because of the contribution from relevant sensitivity tests and the TEM analysis.

## Key Points

- Sparse sampling of Atmospheric Chemistry Experiment Fourier Transform Spectrometer exaggerates the stratospheric nitrous oxide trends
- Transformed Eulerian Mean analysis shows that the residual mean advection contributes to the positive nitrous oxide trend in the Tropics
- The Whole Atmosphere Community‐Climate Model simulates weaker hemispheric asymmetries of the nitrous oxide trends compared to reanalyses

## 1 Introduction

Nitrous oxide (N_2_O) is continuously emitted in the troposphere, with a nearly constant rate of change of 2% per decade, and transported into the stratosphere, where it is destroyed by photodissociation mainly in the Tropics above 35 km (Tian et al., 2020). The atmospheric lifetime of N_2_O is approximately 120 years, which makes it an excellent tracer for stratospheric transport studies (Seinfeld & Pandis, 2016). Within the stratosphere, the lifetime of N_2_O depends also on the solar activity because of its influence on the photolysis rates, with slightly decreased lifetime during solar maxima and increased lifetime during solar minima (Prather et al., 2015).

N_2_O enters the stratosphere in the tropics, and is transported toward higher latitudes by the Brewer‐Dobson Circulation (BDC, Brewer, 1949; Dobson, 1956; Dobson et al., 1929). The BDC is driven by the breaking of tropospheric waves that propagate into the stratosphere (e.g., Charney & Drazin, 1961) and is often separated into an advective component, the residual mean meridional circulation (hereafter residual circulation), and a mixing component (Garny et al., 2014). The residual circulation consists in upwelling in the Tropics, followed by poleward flow and downwelling over the middle and high latitudes (Plumb, 2002). The mixing is a two‐way exchange of mass that, within the stratosphere, occurs mostly on isentropic surfaces, thus, it is mainly quasi‐horizontal (Shepherd, 2007). The BDC has a significant impact in determining the stratospheric distribution of chemical tracers, like ozone and greenhouse gases (e.g., Butchart, 2014), and in maintaining the observed meridional and vertical temperature structure of the stratosphere (Holton, 2004). Long‐term changes in the BDC can have significant impacts on the climate system. One of the most important is the effect on the recovery of stratospheric ozone, as a changing BDC would result in changes of its meridional distribution (e.g., Dhomse et al., 2018; Shepherd, 2008). Changes in the BDC also impact the lifetime of Ozone Depleting Substances (ODS) in the stratosphere (Butchart & Scaife, 2001; Waugh & Hall, 2002), as well as the water vapor entering the stratosphere through the Tropics (e.g., Randel & Park, 2019). The troposphere is also affected by BDC changes because of the impact on the mass exchange with the stratosphere (e.g., ozone, Meul et al., 2018), and on the ultra‐violet radiation reaching the surface (Meul et al., 2016).

Given the relevance of the BDC changes, understanding them is thus fundamental to fully comprehend the past and future evolution of climate. Simulations by Chemistry‐Climate Models (CCMs) robustly project an acceleration of the BDC throughout the stratosphere in recent and coming decades due to the increase of greenhouse gases (e.g., Abalos et al., 2021). On the other hand, Oberländer‐Hayn et al. (2016) argue that the global BDC trends in the lower stratosphere in CCMs are caused to a large extent by a lift of the tropopause level in response to global warming rather than an actual speedup of the circulation. Another significant impact of the increase of greenhouse gases is the shrinkage of the stratosphere, that is, the combination of the tropopause rise and the downward shift of the height of the pressure levels above 55 km, that results from its cooling over the last decades (Pisoft et al., 2021). Modeling studies have shown that this stratospheric shrinking can impact the BDC and modulate its changes over the past decades (Eichinger & Šácha, 2020; Šácha et al., 2019). Such modulation consists in a BDC acceleration similar to that resulting from the impact of the tropopause lift (Eichinger & Šácha, 2020). In addition, CCMs simulations show that also the vertical and meridional structure of the BDC has changed in the past decades in response to climate change (Hardiman et al., 2014). Other modeling studies have shown that mixing, both on resolved and unresolved scales, also plays an important role in the simulated magnitudes of the BDC changes in addition to changes in the residual circulation among CCMs (e.g., Eichinger et al., 2019). Recent studies have also shown that ODS, through their impact on ozone, play a significant role in the modeled BDC changes (Abalos et al., 2019). In particular, the ODS decrease resulting from the Montreal Protocol, will reduce the global warming‐induced acceleration of the BDC and potentially lead to hemispheric asymmetries in the BDC trends (Polvani et al., 2019).

The BDC and its changes cannot be measured directly (e.g., Minschwaner et al., 2016), but can be indirectly examined from measurements of stratospheric long‐lived tracers (e.g., Engel et al., 2009; Hegglin et al., 2014) or temperature (Fu et al., 2015). Recently, Strahan et al. (2020) used ground‐based observations of nitric acid and hydrogen chloride to investigate hemispheric‐dependent BDC changes in the stratosphere. Similarly, space‐borne observations of stratospheric tracers are often used to investigate decadal changes in the BDC using, for example, hydrogen fluoride (Harrison et al., 2016), ozone (Nedoluha et al., 2015), or N_2_O (Han et al., 2019). Measurements of stratospheric tracers are often used to calculate the mean Age of Air (AoA, Hall & Plumb, 1994). The mean AoA is a widely used diagnostic for stratospheric transport and is defined as the transit time of an air parcel from the tropical tropopause (or the surface, depending on the definition) to a certain point of the stratosphere (Waugh & Hall, 2002). Engel et al. (2017) used balloon‐borne observations of carbon dioxide and methane to derive mean AoA trends above the northern mid‐latitudes in the mid‐lower stratosphere. Engel et al. (2017) found positive but not statistically significant mean AoA trends over about 40 years (corresponding to a possible slowdown of the BDC), which is in contrast with the modeling studies that simulate a significant acceleration of the BDC over the same region (e.g., Abalos et al., 2021). These discrepancies can be partly attributed to the temporal and spatial sparseness of the measurements and to uncertainties in the mean AoA trends derived from real tracers (Fritsch et al., 2020; Garcia et al., 2011). In addition to ground‐based measurements, space‐borne observations have been used to compute mean AoA trends as well (e.g., Haenel et al., 2015; Stiller et al., 2012). These observational studies using remote sensing measurements have shown a hemispheric asymmetry in the mean AoA trends over 2002–2012, with positive changes in the Northern Hemisphere (NH) and negative changes in the Southern Hemisphere (SH) (e.g., Mahieu et al., 2014; Stiller et al., 2017). The mean AoA indirectly obtained from satellite measurements in these studies does not allow the separation between residual circulation and mixing, which was proven to be important in CCMs (Dietmüller et al., 2018). However, Linz et al. (2021) showed that the effect of mixing can be explicitly calculated using AoA vertical gradients from both models and satellite measurements. In addition, Von Clarmann and Grabowski (2021) (similarly to the early study of Holton & Choi, 1988) proposed an alternative method to infer the stratospheric circulation from satellite measurements of long‐lived tracers by a direct inversion of the continuity equation.

Reanalysis datasets try to fill the gap between observations and free‐running models, providing a global multi‐decadal and continuous state of the past atmosphere by assimilating available observations. Dynamical fields from reanalyses can be used to drive Chemistry‐Transport Models (CTMs) to simulate the distribution of real and synthetic tracers in the atmosphere. In the past decade, these CTM experiments have been used to investigate BDC changes in reanalyses using the AoA diagnostic (e.g., Diallo et al., 2012; Monge‐Sanz et al., 2012; Ploeger et al., 2015). However, significant differences exist in the BDC changes obtained from different reanalyses, both over multi‐decadal and decadal time scales (e.g., Abalos et al., 2015; Chabrillat et al., 2018). Furthermore, the computation of mean AoA largely depends on whether the kinematic velocities or the heating rates are used to drive the CTMs, leading to significant differences within the same reanalysis (Ploeger et al., 2019).

This study is based on the work performed by Minganti et al. (2020, hereafter M2020), who evaluated the climatological BDC in the Whole Atmosphere Community Climate Model (WACCM) version 4 (Garcia et al., 2017). The evaluation in M2020 consisted in studying the impact of the BDC on the climatologies of the stratospheric N_2_O abundancies and of the N_2_O Transformed Eulerian Mean (TEM) budget (Andrews et al., 1987). This evaluation was performed by comparison with simulations of the Belgian Assimilation System for Chemical ObsErvation Chemistry‐Transport Model CTM (BASCOE CTM, Chabrillat et al., 2018) driven by dynamical reanalyses and with the BASCOE reanalysis of Aura Microwave Limb Sounder (MLS) version 2 (BRAM2, Errera et al., 2019). The TEM diagnostic was included in M2020 because it allows separating the effects of transport and chemistry on the rate of change of a stratospheric tracer such as N_2_O (Randel et al., 1994). Within the TEM framework, the impact of transport can be further separated into the impact from the residual circulation and mixing, as was done for ozone and carbon monoxide in Abalos et al. (2013). It is important to note that the mixing obtained from the TEM analysis generally includes contributions from advective transport that are not represented by the residual circulation (Holton, 2004). After studying the climatologies in M2020, the present study aims to evaluate the changing BDC in WACCM in its versions 4 and 6 (Gettelman et al., 2019) by studying multi‐decadal and decadal changes of N_2_O in the stratosphere, comparing them with ground‐based and space‐borne observations and BASCOE CTM simulations. We also evaluate the changes in TEM N_2_O budget in WACCM and in the BASCOE CTM. We compare the model simulations with ground‐based observations of N_2_O from Fourier transform infrared (FTIR) spectrometers that are part of the Network for the Detection of Atmospheric Composition Change (NDACC) at four stations in the SH and NH subtropics as well as at mid‐latitudes (De Mazière et al., 2018, http://www.ndaccdemo.org/). We also use satellite measurements from the Atmospheric Chemistry Experiment Fourier Transform Spectrometer (ACE‐FTS, Bernath et al., 2021). Contrary to M2020, we cannot use N_2_O from BRAM2 because of the unrealistic negative drift in the MLS N_2_O dataset (Livesey et al., 2021). The BASCOE CTM is driven by four modern reanalyses that are part of the SPARC (Stratosphere‐troposphere Processes and their Role in Climate) Reanalysis Intercomparison Project (S‐RIP, Fujiwara et al., 2017).

The present study is structured as follows. Section 2 describes the observational and modeling datasets used in this study, as well as the TEM diagnostics and the regression model used to derive linear trends. In Section 3, we use FTIR observations to evaluate the trends in the stratospheric N_2_O columns obtained from WACCM and the CTM simulations and from satellite measurements. In Section 4, using ACE‐FTS as a reference, we study the global N_2_O trends in the stratosphere and focus on the differences in the trend patterns among datasets. In Section 5, we investigate the N_2_O TEM budget from WACCM version 6 and a BASCOE simulation in order to separate the impact of the residual circulation and mixing on the N_2_O trends. Finally, Section 6 concludes the study with a summary of the principal findings.

## 2 Data and Methods

This section describes the observational and model data as well as the methods used in this study (see Tables 1 and 2). Throughout the study, we will refer to the CCMs and the BASCOE CTM simulations as ”models” to distinguish them from the observations obtained from the FTIR and ACE‐FTS. For the sake of brevity, we refer to M2020 for a more detailed description of the dataset (BASCOE CTM, WACCM version 4, and S‐RIP reanalyses) and methods (TEM framework) already used there.

**Table 1:** Overview of the Models and Satellite Measurements Used in This Study

Dataset name	Full Name	Reference	Year range	Vert. resol. + top
WACCM‐REFC1	Whole Atmosphere Community Climate Model	Marsh et al. (2013); Garcia et al. (2017)	1985–2018	L66, 5.96 10^−6^ hPa
WACCM‐REFD1	Whole Atmosphere Community Climate Model	Gettelman et al. (2019)	1985–2018	L70, 5.96 10^−6^ hPa
CTM + ERAI	ECMWF Reanalysis Interim	Dee et al. (2011)	1985–2018	L60, 0.1 hPa
CTM + ERA5	ECMWF Reanalysis 5	Hersbach et al. (2020)	1985–2019	L86, 0.01 hPa
CTM + JRA55	Japanese 55‐year Reanalysis	Kobayashi et al. (2015)	1985–2018	L60, 0.2 hPa
CTM + MERRA2	Modern‐Era Retrospective analysis for Research and Applications	Gelaro et al. (2017)	1985–2018	L72, 0.01 hPa
ACE‐FTS	Atmospheric Chemistry Experiment Fourier Transform Spectrometer	Bernath et al. (2021)	2005‐present	L42, 150 km

**Table 2:** Overview of FTIR Stations Considered in This Study

Station name	Reference	Location (lat and lon)	Altitude	strato DOFS
Lauder	Zhou et al. (2019)	45.4°S and 169.68°E	370 m	2
Wollongong	Griffith et al. (2012)	34.45°S and 150.88°E	30 m	2
Izaña	García et al. (2021)	28.30°N and 16.48°E	2367 m	1.5
Jungfraujoch	Zander et al. (2008)	46.55°N and 7.98°E	3580 m	1.1

### 2.1 Ground‐Based FTIR Observations

We use ground‐based measurements of stratospheric N_2_O columns obtained at four stations that are part of NDACC: Lauder (New Zealand, 45°S), Wollongong (Australia, 34°S), Izaña (Spain, 28°N), and Jungfraujoch (Switzerland, 46°N) (Zhou et al., 2019). The solar absorption spectra under clear‐sky conditions with the ground‐based FTIR measurements taken under the auspices of the NDACC allow the acquisition of long‐term consistent data sets. The stations have been chosen at the mid‐latitudes and subtropics where the observed BDC changes are the largest (e.g., Strahan et al., 2020).

At Jungfraujoch, measurements have been obtained from two spectrometers: an instrument developed at the University of Liège (1984–2008), and a Bruker IFS 120HR (early 1990’s‐present) (Prignon et al., 2019; Zander et al., 2008). In this study, we use the spectra taken by the Bruker spectrometer to investigate the most recent period. Ground‐based measurements of N_2_O profiles at Lauder started in 2001 with a Bruker 120HR spectrometer, replaced in 2018 (with 6 months overlap) by a Bruker 125HR (Strong et al., 2008; Zhou et al., 2019). The Lauder station is particularly relevant as is the only FTIR site of NDACC located in the SH mid‐latitudes. The Wollongong station has provided data for the SH subtropics since 1996. Solar spectra were measured with a Bomem instrument until 2007, which was then replaced by a Bruker 125HR (Griffith et al., 2012). N_2_O profiles are also measured at the Izaña Observatory since 1999. This high‐altitude station is characterized by excellent conditions for FTIR spectroscopy, with clear sky conditions for most of the year. Observations started using a Bruker 120M spectrometer and continued, since 2005, with a Bruker 125HR (García et al., 2021). The retrieval code for the N_2_O profiles is the SFIT‐v4 (v0.9.4.4) for the Jungfraujoch, Lauder and Wollongong stations, and PROFITT9 for the Izaña station (Zhou et al., 2019).

We consider stratospheric N_2_O columns between 12 and 40 km of altitude because the instruments at all stations are the most sensitive to the measured N_2_O profiles over this stratospheric region (not shown). The degrees of freedom for signal (DOFS), which quantify the vertical resolution of the measurement (Rodgers, 2000), vary largely between the stations. For N_2_O, the stratospheric DOFS between 12 and 40 km of the instruments in the SH are approximately 2, allowing the separation of two layers within the stratosphere. On the other hand, the stratospheric DOFS of the instruments in the NH are around 1.5 for Izaña, and 1 for Jungfraujoch, limiting the analysis to one stratospheric layer between 12 and 40 km. Thus, in order to perform a fair comparison, we compute one stratospheric N_2_O column between 12 and 40 km for all stations. In order to take into account the limited sensitivity of the FTIR measurements, we smooth the ACE‐FTS data and the model output on the FTIR vertical grid using the FTIR averaging kernels as described in Langerock et al. (2015).

### 2.2 Spaceborne Measurements ‐ ACE‐FTS

ACE‐FTS, onboard the SCISAT Canadian satellite, was launched in August 2003 on a high inclination (74°) low earth orbit (650 km) and is still in operation in 2022 (Bernath, 2017; Bernath et al., 2005). The ACE‐FTS instrument measures the infrared absorptions from solar occultations between 2.2 and 13.3 μm with a spectral resolution of 0.02 cm^−1^. This allows the retrieval of vertically resolved mixing ratio profiles for 44 molecules and 24 isotopologues from each measurement (Bernath et al., 2020).

In this study, we use version 4.1 of the ACE‐FTS data. It differs from previous versions by the significantly better retrievals at low altitudes and led to substantially improved trends compared to the earlier version 3.5 (Bernath et al., 2021). For N_2_O, previous comparisons of v3.6 with independent satellite instruments showed a good agreement below 35 km (within 10%) and larger biases above that level (within 20%, Sheese et al., 2017). In our study, N_2_O profiles are filtered for outliers using the method described in Sheese et al. (2017) and are then vertically regridded to a constant pressure vertical grid using a mass‐conservative scheme (Bader et al., 2017). For trend analysis, profiles are monthly averaged on latitude bins with 5°spacing from pole to pole.

In order to compare the trend analysis of model simulations with those obtained by ACE‐FTS, the model datasets are first re‐sampled from their native temporal and spatial grids (model space) to match those of ACE‐FTS (observational space). This is important in particular due to the low sampling of ACE‐FTS—only 30 daily profiles due to the solar occultation method. The re‐sampling is done by finding model output adjacent in time to each ACE‐FTS profile (BASCOE and WACCM datasets used in this study have, respectively, 6 hourly and daily output) and then by linearly interpolating the model values in time and space at the profile geolocation. The re‐sampled model datasets are then averaged over a month as done with ACE‐FTS.

### 2.3 BASCOE CTM and Driving Reanalyses

In this study, we use the BASCOE CTM driven by four dynamical reanalyses: the European Center for Medium‐Range Weather Forecast Interim reanalysis (ERAI, Dee et al., 2011), and its newer version ERA5 (Hersbach et al., 2020), the Modern‐Era Retrospective analysis for Research and Applications version 2 (MERRA2, Gelaro et al., 2017), and the Japanese 55‐year Reanalysis (JRA55, Kobayashi et al., 2015). In the following, we provide a brief overview of the BASCOE CTM and the ERAI, MERRA2 and JRA55 reanalyses, as more detailed information can be found in such companion studies: Chabrillat et al. (2018); Prignon et al. (2019, 2021) and M2020. Since ERA5 is not detailed in these publications, we provide a more detailed description.

The BASCOE CTM is built on a kinematic transport module (that takes as input the surface pressure and the horizontal winds) with a flux‐form semi‐Lagrangian (FFSL) advection scheme (Lin & Rood, 1996). The FFSL scheme is run on a common horizontal grid of 2° × 2.5° for all the reanalyses, while the vertical grid depends on the input reanalysis. The chemical scheme explicitly solves for stratospheric chemistry, and includes 65 chemical species and 243 reactions (Prignon et al., 2019). ERAI and JRA55 have 60 levels up to 0.1 hPa, MERRA2 has 72 levels up to 0.01 hPa. The model setup, as well as the boundary conditions (including those for N_2_O), are the ones used in Prignon et al. (2019), M2020 and Prignon et al. (2021). Readers are directed toward Chabrillat et al. (2018) for a detailed description of the BASCOE CTM and its driving by the ERAI, JRA55 and MERRA2 reanalyses.

The ERA5 reanalysis is the fifth generation of reanalysis produced by the ECMWF and covers the 1979‐present period, with a programmed extension back to 1950 (Hersbach et al., 2020). The horizontal resolution is 31 km, with hourly output frequency, and the vertical grid ranges from the surface to 0.01 hPa with 137 levels and with 300–600 m vertical spacing in the troposphere and stratosphere, which increases to 1–3 km above 30 km. ERA5 suffers from a cold bias in the lower stratosphere from 2000 to 2006. For this reason, a new analysis (ERA5.1) has been produced for that period to correct for that bias (Simmons et al., 2020). In this study, the BASCOE CTM was driven by ERA5.1 for the 2000–2006 period. For computational reasons, the vertical resolution is reduced to 86 levels from the original 137 keeping the original vertical spacing in the stratosphere, and we used 6‐hourly (0000, 0600, 1200, 1800 UTC) data. As done for the other reanalyses, the ERA5 data on the fine 31‐km grid were truncated at wavenumber 47 to avoid aliasing on the target 2.5° × 2°horizontal grid (Chabrillat et al., 2018).

In order to further investigate the contribution of transport in ERA5, we performed two sensitivity tests with the BASCOE CTM driven by that reanalysis. To isolate the contribution of transport, the first sensitivity test consists of a fixed N_2_O run, that is, a BASCOE CTM simulation where N_2_O does not increase over time. We accomplished that by performing a BASCOE CTM run exactly as the ERA5 simulation but keeping the N_2_O volume mixing ratios at the surface fixed to their values at the beginning of the simulation (cst‐N_2_O). Any N_2_O trend for the cst‐N_2_O simulation is therefore due only to the effect of transport. The second sensitivity test is a perpetual year simulation that is complementary to cst‐N_2_O, and consists of an experiment where the transport does not change over time (cst‐dyn). In order to include a complete Quasi Biennial Oscillation cycle (QBO, Baldwin et al., 2001), we used the years 2006 and 2007 from ERA5.1 and ERA5, respectively. Those years are unusual (but convenient) because the QBO lasted exactly 24 months (see the zonal wind data at Singapore https://www.geo.fu-berlin.de/met/ag/strat/produkte/qbo/singapore.dat). We used the dynamics of the year 2006 to simulate even years and from the year 2007 for odd years. All the N_2_O changes simulated by cst‐dyn are due to its constant increase at the surface.

### 2.4 WACCM

In this study, we use two versions of WACCM: version 4 (Garcia et al., 2017; Marsh et al., 2013) and version 6 (Gettelman et al., 2019). WACCM version 4 (WACCM4) is the atmospheric component of the Community Earth System Model version 1.2.2 (CESM, Hurrell et al., 2013), which has been developed by the U.S. National Center of Atmospheric Research. It is the extended (whole atmosphere) version of the Community Atmosphere Model version 4 (CAM4, Neale et al., 2013). WACCM4 has a longitude‐latitude grid of 2.5° × 1.9°and 66 vertical levels from the surface to about 140 km altitude, with 1.1–1.75 km vertical spacing in the stratosphere. The physics of WACCM4 is the same as CAM4 and the dynamical core is a finite volume with a horizontal discretization based on a conservative flux‐form semi Lagrangian (FFSL) scheme (Lin, 2004). WACCM4 is not able to internally generate the QBO; thus, it is nudged toward observations of stratospheric winds (Matthes et al., 2010). In this study, we use the WACCM4 version included within the SPARC (Stratosphere‐troposphere Processes And their Role in Climate) Chemistry‐Climate Model Intercomparison Phase 1 (CCMI‐1, Morgenstern et al., 2017). In particular, we use the REFC1 experiments (WACCM‐REFC1), which consist of simulations of the recent past (1960–2018) using state‐of‐the‐art historical forcings and observed sea‐surface temperatures (Morgenstern et al., 2017). For N_2_O, the boundary conditions are prescribed using the forcing recommended by the CCMI (Eyring et al., 2013). Compared to the default WACCM4 version, WACCM‐REFC1 includes important modifications of the treatment of heterogeneous chemistry and of the gravity waves parameterization, which ultimately improve the simulation of ozone in the SH (Garcia et al., 2017). In this study, we use three realizations of the WACCM‐REFC1 configuration for the 1985–2018 period.

Version six of WACCM (WACCM6) is the extension to the whole atmosphere of version six of CAM that is part of version two of CESM (Danabasoglu et al., 2020). The default horizontal resolution of WACCM6 is 0.9° × 1.25° latitude‐longitude, with 70 levels in the vertical from the ground to around 140 km, with vertical resolution similar to WACCM4. The transition from WACCM4 to WACCM6 involved several changes in the physics and chemistry that are described in Gettelman et al. (2019). WACCM6 is part of the Coupled Model Intercomparison Project Phase 6 (CMIP6, Eyring et al., 2016), and is used in the CCMI‐2022 activity (i.e., the successor of CCMI‐1, Plummer et al., 2021). Within CCMI‐2022, we use the REFD1 WACCM6 experiments (WACCM‐REFD1), that is, a suite of hindcast experiments for the recent past (1960–2018) used to compare with observations. The REFD1 experiments use the databases for historical forcings and observed sea surface temperatures developed for the CMIP6. The N_2_O emissions are specified following the CMPI6 recommendation for historical simulations, that is, following Meinshausen et al. (2020). Although WACCM6 can internally produce the QBO, the REFD1 experiments require a nudged QBO toward observed winds to ensure synchronization with historical variability. In this study, we use one realization of the WACCM‐REFD1 experiments for the 1985–2018 period.

### 2.5 TEM Diagnostics

For stratospheric tracers, the TEM diagnostics (Andrews et al., 1987) allows separating the impact of transport and chemistry on the zonal mean local rate of change of a tracer with mixing ratio χ:

(1) $${\bar{\chi }}_{t}=-v^{*}{\bar{\chi }}_{y}-w^{*}{\bar{\chi }}_{z}+e^{z/H}\nabla \cdot M+\bar{S}+\bar{\epsilon },$$

where *χ* represents N_2_O, $M=-e^{-z/H}\left(\bar{v^{\prime }\chi^{\prime }}-\bar{v^{\prime }\theta^{\prime }}{\bar{\chi }}_{z}/{\bar{\theta }}_{z},\bar{w^{\prime }\chi^{\prime }}+\bar{v^{\prime }\theta^{\prime }}{\bar{\chi }}_{y}/{\bar{\theta }}_{z}\right)$ is the eddy flux vector, and (v*, w*) are the meridional and vertical components of the residual circulation, respectively. Overbars denote zonal means and prime quantities indicate deviations from it, while subscripts indicate partial derivatives. *H* = 7 km is the scale height, and *z* ≡ − *Hlog*_*e*_(*p*/*p*_*s*_) is the log‐pressure altitude, with the surface pressure *p*_*s*_ = 10^5^ Pa. The *S* term is the net rate of change due to chemistry, defined as the difference between the production $\left(\bar{P}\right)$ and loss $\left(\bar{L}\right)$ rates $\bar{S}=\bar{P}-\bar{L}$. The $\bar{\epsilon }$ contribution represents the residual of the budget, that is, the difference between the actual rate of change of $\bar{\chi }$ and the sum of the transport and chemistry terms on the right‐side hand of Equation 1.

The transport terms in Equation 1 can be grouped as follows:

(2) $${\bar{\chi }}_{t}=ADV+MIX+\bar{S}+\bar{\epsilon },$$

where $ADV=\left(-v^{*}{\bar{\chi }}_{y}-w^{*}{\bar{\chi }}_{z}\right)$ and *MIX* = *e* ^*z*/*H*^ **∇ ⋅ M** represent the contribution of the residual circulation and of the resolved mixing, respectively. We refer to M2020 for a more detailed description of the TEM framework applied to the N_2_O mixing ratios in the stratosphere and for a comprehensive discussion of the contribution of each term to the N_2_O budget.

### 2.6 Derivation of Trends With the Dynamical Linear Modeling Tool

In this study, we investigate decadal trends using the Dynamical Linear Modeling regression tool (DLM, Alsing, 2019). DLM is based on Bayesian inference and provides a number of possible models to analyze time series. Each model is characterized by some unknown parameters, and the DLM computes the posterior probability distribution of those parameters using a combination of Kalman filtering and Markov chain Monte Carlo method.

For a given atmospheric time‐series *y*_*t*_, a generic DLM model is composed of four components: a linear background trend, a seasonal cycle with 12‐ and 6‐months periods, forcing terms described by a number of regressor variables and an auto‐regressive component:

(3) $$\begin{array}{ccc} y_{t} & = & \beta_{1,t}z_{1,t}+\beta_{2,t}z_{2,t}\ldots +\beta_{n,t}z_{n,t}\\ & & +\;\beta_{1,t}^{12}\sin\left(2\pi t/12\right)+\beta_{2,t}^{12}\cos\left(2\pi t/12\right)\\ & & +\;\beta_{1,t}^{6}\sin\left(2\pi t/6\right)+\beta_{2,t}^{6}\cos\left(2\pi t/6\right)\\ & & +\;\mu_{t}\\ & & +\;z_{t}^{AR}\\ & & +\;\epsilon_{t}. \end{array}$$

In Equation 3, the terms *β*_*i*,*t*_ *z*_*i*,*t*_ represent the contribution to *y*_*t*_ from the regressors, where *z*_*i*,*t*_ is the corresponding time‐series for each regressor. The 6‐ and 12‐month seasonal cycles are modeled respectively by $\beta_{1,t}^{6}\sin\left(2\pi t/6\right)+\beta_{2,t}^{6}\cos\left(2\pi t/6\right)$ and $\beta_{1,t}^{12}\sin\left(2\pi t/12\right)+\beta_{2,t}^{12}\cos\left(2\pi t/12\right)$. The *μ*_*t*_ term denotes the linear fit term, and $z_{t}^{AR}$ the auto‐regressive term, defined similarly to the Cochrane‐Orcutt correction (Kyrölä et al., 2013), and *ϵ*_*t*_ is the uncertainty.

Contrarily to a multi‐linear regression (MLR) model, the background linear fit *μ*_*t*_ and the amplitudes of the seasonal cycles $\beta_{i,t}^{6,12}$ in DLM can vary with time (i.e., they are non‐parametric). Their degrees of time‐dependence are the unknown model parameters and are initially set by the user and inferred from the data during the model run. Furthermore, the auto‐regressive process in the DLM is computed within the model run together with the other parameters, not as a post‐run correction as done in the MLR, and its uncertainties are carefully taken into account within the error propagation. In addition, the standard DLM implementation has time‐varying (heteroscedastic) uncertainty distribution, when time‐varying uncertainties are available. DLM was recently used to investigate stratospheric ozone trends in observations and models (Ball et al., 2017, 2018). A more detailed description of the DLM models and their implementation can be found in Laine et al. (2014). For a more comprehensive review of time‐series analysis using DLM, refer to Durbin and Koopman (2012).

As regressor variables, we used the 30 cm radio flux as a solar proxy (de Wit et al., 2014), an index for the El‐Nino Southern Oscillation (Wolter & Timlin, 2011) from the National Oceanic and Atmospheric Administration (http://www.esrl.noaa.gov/psd/enso/mei/), and two indices for the QBO at 30 and 50 hPa from the Freie Universität Berlin (http://www.geo.fu-berlin.de/en/met/ag/strat/produkte/qbo/index.html). We fed the DLM model with monthly data, running 3,000 samples where the first 1,000 were considered as a warmup and discarded. We also tried 10,000 realizations and 3,000 as warmup with very similar results (not shown). We performed several sensitivity tests to determine the appropriate values of the initial model parameters, that is, the degree of time‐dependence of the linear trend and seasonal cycles, in order to allow a reasonable time‐dependence without being unrealistic. The different combinations of these values did not provide significant differences, so we kept the recommended values.

The linear trends are computed from the distribution of the fit samples *μ*_*t*_ as the difference between the end and start dates of the considered period (delta = *μ*_*t*_[*end*] − *μ*_*t*_[*start*]), weighted by the number of the years. From the resulting delta distribution, the uncertainties associated with the trend are computed as the percentage of its positive (negative) values. This percentage can be interpreted as the posterior probability that the trend is positive (negative) between the considered dates. In this way, we do not make any assumption on the shape of the distribution of the trends.

## 3 Stratospheric N_2_O Columns and Their Trends

**Figure 1:**
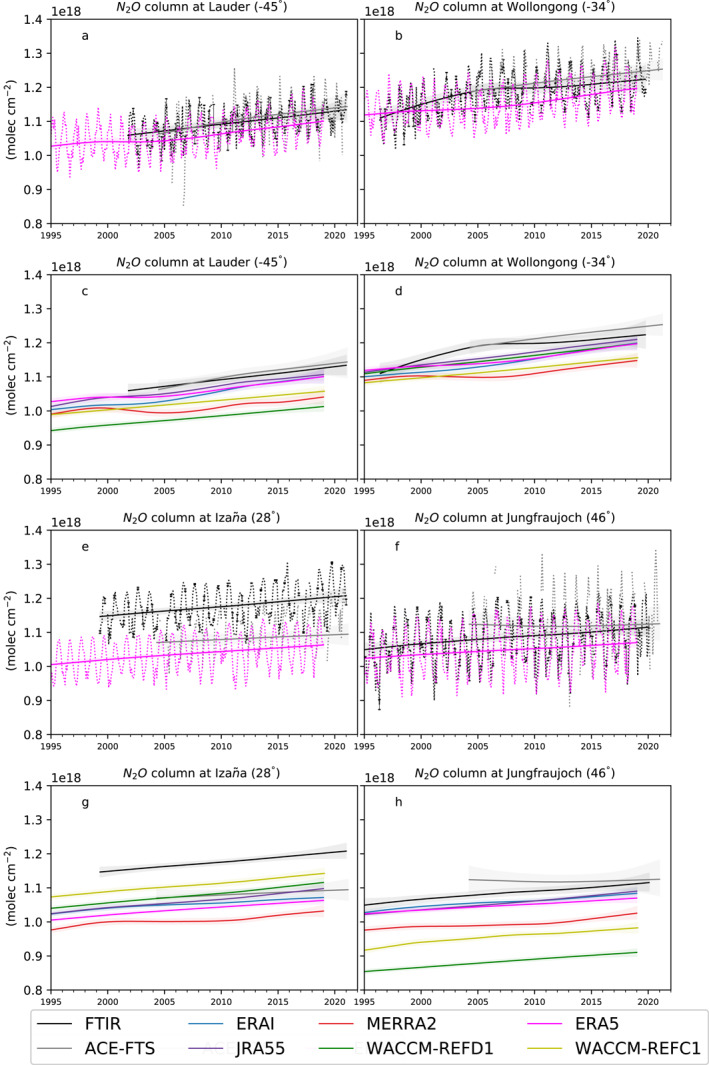
Time‐series of stratospheric N_2_O columns (12–40 km) from observations and models at four stations. Continuous lines show the linear fit obtained by the DLM regression, dashed lines depict the N_2_O column data. The color code is shown in the legend. The vertical error bars in panels a, b, e, f represent the standard error of the monthly mean. Panels a, b show Lauder, panels b,d show Wollongong, panels e,g show Izaña and panels f,h show Jungfraujoch. Panels a, b, e, f: DLM fits and data for FTIR and ACE‐FTS measurements and the BASCOE simulation driven by ERA5. Panels c, d, g, h: DLM fits for all the datasets considered. The model and satellite data are interpolated to the longitude and latitude of the station, and vertically regridded to match the retrieval layering schemes. After the regridding, the data were smoothed using the FTIR averaging kernels. The colored shadings represent the uncertainties from the 2.5 and 97.5 percentiles of the distributions from the DLM.

Figure 1 shows the linear fits of the monthly stratospheric N_2_O columns (12–40 km) at the four FTIR stations, together with the initial N_2_O columns from the observations and the ERA5 simulation. In this analysis, we do not apply the FTIR time sampling to the model output because sensitivity tests using WACCM‐REFD1 at each station showed no significant impact of the FTIR time sampling on the recovered trends of the N_2_O columns (not shown). The stratospheric N_2_O columns computed between 12 and 40 km of altitude are highly sensitive to the N_2_O increase in the lower stratosphere, which is mainly the result of the continuous growth in the troposphere (Tian et al., 2020) and can also be impacted by structural changes of the atmosphere (e.g., the global rise of the tropopause, Xian & Homeyer, 2019). Consequently, all datasets exhibit an increase in the stratospheric N_2_O columns over the last two decades.

Above Lauder, the linear fit of the stratospheric N_2_O columns from the ERA5 simulation is in agreement with the observations, similarly to JRA55 and ERAI. WACCM‐REFD1 underestimates the stratospheric N_2_O columns compared to the observations by around 10%, and performs worse than its earlier version WACCM‐REFC1, which differs from the observations by only 5%. At Wollongong, the slope of the linear fit of the N_2_O columns measured by the FTIR, and to a lesser extent by ACE‐FTS, is steeper before 2005 compared to the following period. This change of gradient is not visible in any of the model simulations. On the contrary, some of the models show a slower increase before 2005, followed by a more rapid increase (e.g., the ERA5 simulation). Contrarily to Lauder, the WACCM‐REFD1 simulation delivers more realistic stratospheric N_2_O columns compared to its previous version WACCM‐REFC1.

Above Izaña, all the models underestimate the stratospheric N_2_O columns with respect to the FTIR observations, with the largest difference reaching 14% for MERRA2. Concerning ACE‐FTS, the bias with FTIR measurements is around 8%, which is qualitatively consistent with the results of Strong et al. (2008), even though they used v2.2 of ACE‐FTS. However, García et al. (2021) showed good agreement above Izaña for tropospheric N_2_O abundances and total N_2_O columns obtained from independent measurements. The difference between the stratospheric N_2_O columns measured by FTIR and ACE‐FTS could be explained by the poor coverage of ACE‐FTS over the tropical and subtropical regions. Since the ACE‐FTS measurements represent a latitude band, the observed N_2_O results biased toward the values measured at higher latitudes, where more occultations are available (Kolonjari et al., 2018). Since the N_2_O abundances decrease poleward (Jin et al., 2009), this could explain the low bias in the stratospheric N_2_O columns measured by ACE‐FTS compared to those obtained from FTIR.

Above Jungfraujoch, there is the largest spread in the linear fits of the stratospheric N_2_O columns, with the largest differences reaching around 25% between ACE‐FTS and WACCM‐REFD1. Prignon et al. (2019) compared lower stratospheric columns of chlorodifluoromethane (HCFC‐22) between an earlier WACCM version and FTIR measurements, and showed that WACCM consistently underestimates the HCFC‐22 columns compared to the FTIR measurements. Since both N_2_O and HCFC‐22 (which has an atmospheric lifetime of 12 years, Prignon et al., 2019) are produced at the surface and transported into the stratosphere, this underestimation in WACCM could indicate a shortcoming in simulating the accumulation of long‐lived tracers in the stratosphere above the northern mid‐latitudes. Indeed, Angelbratt et al. (2011) already highlighted that the stratospheric transport has a large impact on the N_2_O columns above Jungfraujoch compared to stations at higher latitudes. Regarding the observational datasets, there is a considerable disagreement between the FTIR instrument and ACE‐FTS before 2012, showing increasing and decreasing N_2_O columns, respectively. This is in contrast with the remarkably good agreement in the SH between the two datasets. This difference between the stratospheric N_2_O columns in ACE‐FTS and FTIR measurements will be further addressed in Section 4.

In the Tropics and above the lower stratospheric mid‐latitudes, the N_2_O abundances are inversely proportional to the mean AoA (Andrews et al., 2001; Galytska et al., 2019; Strahan et al., 2011). The stratospheric N_2_O columns at mid‐latitudes considered here are highly sensitive to the N_2_O abundances in the lower stratosphere, hence the inverse relationship also holds for the stratospheric N_2_O columns above the mid‐latitudes. Thus, the lower stratospheric N_2_O columns in MERRA2 compared to the other datasets across the stations are consistent with the older mean AoA throughout the stratosphere found using MERRA2 by Chabrillat et al. (2018). The N_2_O distribution in the stratosphere is opposite also to the total inorganic fluorine F_*y*_. N_2_O is emitted in the troposphere while F_*y*_ is produced in the stratosphere, and, as a consequence of the poleward transport of the BDC, N_2_O is removed and F_*y*_ is increased in the stratospheric mid‐latitudes. In the light of this relationship between N_2_O and F_*y*_, the underestimated N_2_O columns above Lauder and Jungfraujoch in MERRA2 are consistent with larger stratospheric F_*y*_ columns in MERRA2 compared to the other reanalyses above those stations (Prignon et al., 2021).

**Figure 2:**
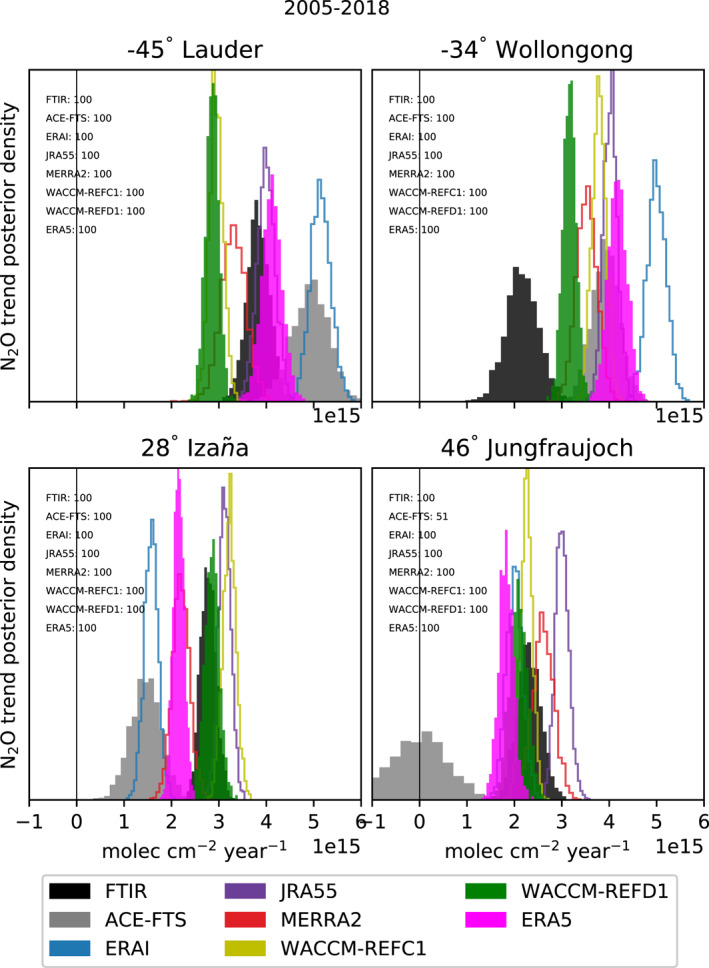
Posterior probability of positive changes of the DLM linear trend of the stratospheric N_2_O columns (12–40 km) for the four FTIR stations (2005–2018). The color code is shown in the legend. For reference, the N_2_O trend in the troposphere (5.5–10.5 km) is approximated from the data in Bernath et al. (2020) as 4.3e15 molec cm^−2^ year^−1^.

Figure 2 shows distributions of the trend of the stratospheric N_2_O columns obtained from the respective linear fits over the common period 2005–2018. The N_2_O trends at the surface have already been compared for a number of FTIR stations (including Lauder, Wollongong and Izaña) against observations from flask samples, showing an excellent agreement (Zhou et al., 2019).

Above Lauder, the N_2_O trends obtained with ERA5 and JRA55 are in good agreement with the FTIR measurements, but are underestimated in WACCM‐REFD1 (around 25%) with no particular improvement with respect to WACCM‐REFC1. The ERAI simulation delivers the largest N_2_O trends, with more than 30% difference with respect to the FTIR measurements. At Wollongong, the N_2_O trend obtained with the FTIR measurements is the smallest because the N_2_O increase above that station is smoother compared to the other datasets. Interestingly, the N_2_O trend simulated by WACCM‐REFD1 is the closest to the trend obtained from the FTIR observations, while the trend obtained with ERA5 is almost twice as large. As for Lauder, the N_2_O trends obtained from ERAI are the largest at this station. Above Izaña, WACCM‐REFD1 agrees remarkably well with the FTIR (difference around 3%), while the trend from ERA5 lies between the trends measured from FTIR and ACE‐FTS, with around 20% difference compared to FTIR. Above Jungfraujoch, the trend in the N_2_O columns from WACCM‐REFD1 agrees with the trend from the FTIR within 10% difference and is similar to what is obtained with ERA5. The largest trends are obtained with MERRA2 and JRA55, reaching 13% and 30% difference compared to the FTIR, respectively. The decreasing N_2_O stratospheric column in ACE‐FTS before 2012 results in a near‐zero trend, which is in contrast with the trends obtained by the other datasets, which approximately range from 2 to 3 × 10^15^ molec cm^−2^ year ^−1^.

Considering decadal changes, the observations and the ERA5 and ERAI simulations show larger trends of the stratospheric N_2_O columns in the SH than in the NH, especially at mid‐latitudes (respectively, Lauder and Jungfraujoch). WACCM‐REFD1 also shows this hemispheric difference at mid‐latitudes, which is a clear improvement with respect to WACCM‐REFC1. Those asymmetries are consistent with the results of Strahan et al. (2020), who found significantly negative mean AoA trends in the SH compared to the NH using HCl and HNO_3_ measured at several ground‐based FTIR stations. In addition, the hemispheric differences of the N_2_O trends are also consistent with the results of Prignon et al. (2021), who found larger and more significant F_*y*_ trends from FTIR above Jungfraujoch than above Lauder.

We conclude the section by providing a short description of the limits of using stratospheric columns of N_2_O from FTIR measurements. As mentioned earlier, the stratospheric N_2_O columns between 12 and 40 km are primarily influenced by the steady increase in the lowermost stratosphere below 15 km. The DOFS of the FTIR instrument at Jungfraujoch for the stratosphere (12–40 km) is close to 1.1. Thus, the FTIR measurements at that station cannot resolve more than one partial column between 12 and 40 km, which can hinder the detection of N_2_O trends in the middle and upper stratosphere (i.e., above 30 km) because of the influence of the increase in the lowermost stratosphere. Indeed, it was shown that stratospheric N_2_O trends over the last decades, obtained both from satellite measurements and model simulations, do not consist of just a global increase, but largely depend on latitude and height (e.g., Froidevaux et al., 2019). Therefore, we will consider latitudinal‐ and vertical‐dependent trends of N_2_O mixing ratios in the following section.

## 4 Global N_2_O Linear Trends

### 4.1 Trends in the ACE‐FTS Observational Space

**Figure 3:**
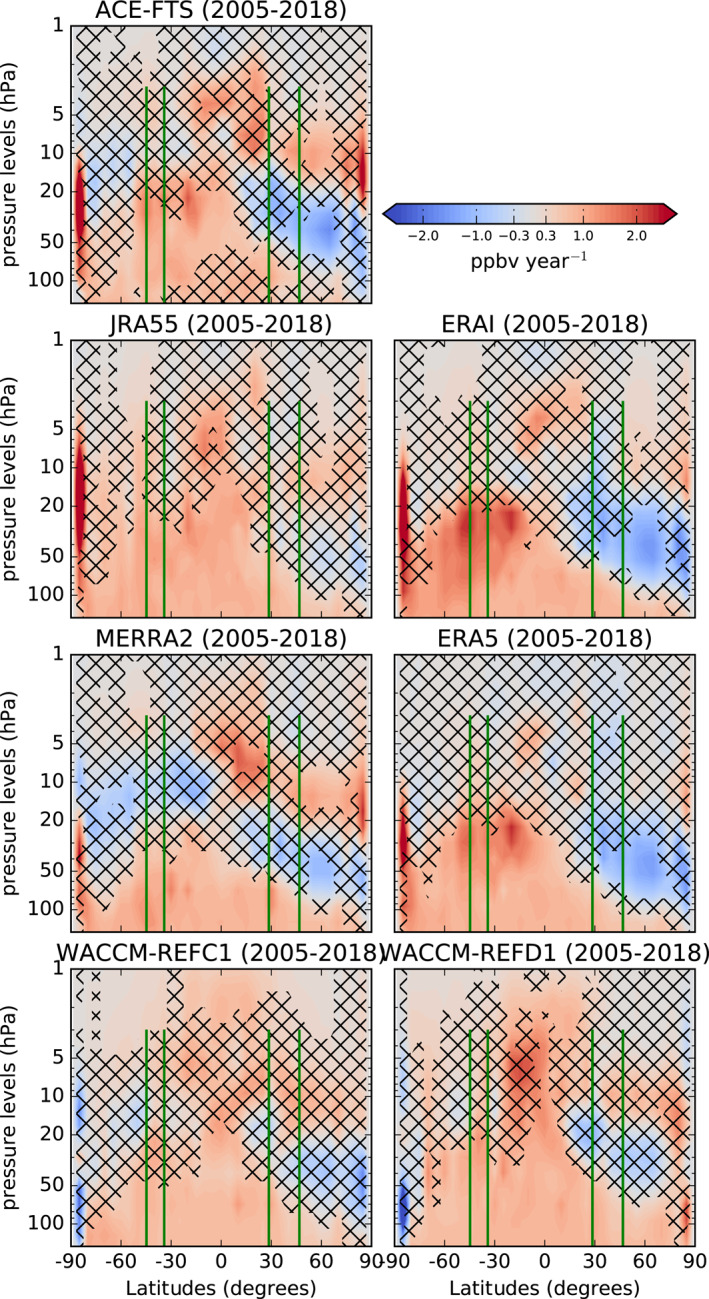
Latitude‐pressure cross‐sections of N_2_O linear trends (pptv year^−1^) obtained from the DLM (2005–2018). The N_2_O simulated by the model is interpolated to the location and timing of the observations, see text for details. The black crosses indicate grid‐points where the probability of positive/negative N_2_O changes is smaller than 95%. The green vertical lines identify the position of the FTIR stations together with their vertical coverage.

Figure 3 shows latitude‐vertical cross sections of the linear trends of the N_2_O mixing ratios for the various datasets, over the 2005–2018 period. In order to reduce the sampling bias, the model datasets are sampled in space and time as the ACE‐FTS measurements before the computation of the trends. We use the ACE‐FTS measurements as a reference, because they encompass this period with global coverage and good stability (Bernath et al., 2020, 2021).

In the upper stratosphere above 10 hPa, the N_2_O trends from ACE‐FTS are positive, with larger trends in the NH that are found significant at lower levels than in the SH. The ERAI‐driven simulation qualitatively reproduces these patterns in the upper stratosphere, while the other model datasets differ from ACE‐FTS, especially ERA5. A common feature among all datasets is an increase in N_2_O above the Equator in the upper stratosphere, around 5 hPa. At those altitudes of the tropical pipe, the upward transport of N_2_O by the residual circulation reaches its maximum (see M2020).

In the mid‐lower stratosphere below 20 hPa, ACE‐FTS shows a clear hemispherical asymmetry (meridional dipole) in the N_2_O trends, with significantly negative values in the NH and significantly positive in the SH. Above the location of Jungfraujoch (the most northern vertical green line), the negative N_2_O trend detected by ACE‐FTS in the mid‐lower stratosphere is responsible for the disagreement with the FTIR observations discussed in the previous section, as the layer of the stratospheric N_2_O column encompasses regions of both positive (lowermost and upper stratosphere) and negative (mid‐lower stratosphere) N_2_O trends. The meridional dipole is significant also over a shorter period (2005–2012, not shown) and corroborates a number of previous findings over that period using satellite measurements of HCl (Mahieu et al., 2014) and mean AoA derived from space‐borne measurements of SF_6_ (Haenel et al., 2015). In regions where the N_2_O abundances are larger than 100 ppbv, that is, approximately below 10 hPa, the N_2_O linear trends are opposite to those obtained with its product NO_2_, because the two tracers are correlated by an inverse linear relationship (Plumb & Ko, 1992). Below 20 hPa, the N_2_O meridional dipole from ACE‐FTS is consistent with the pattern of the decadal trends of NO_2_ obtained from independent satellite measurements (Dubé et al., 2020; Galytska et al., 2019).

The meridional dipole in the N_2_O trends derived from ACE‐FTS is generally reproduced by the CTM simulations, with ERAI and ERA5 delivering trends that are most similar to the satellite measurements. Prignon et al. (2021) used the same simulations as the present study to investigate global stratospheric trends of total inorganic fluorine F_*y*_. The dipoles obtained here in the N_2_O trends from the ECMWF reanalyses are consistent with the opposite trends of F_*y*_ for almost the same period (Prignon et al., 2021). For WACCM, the strength of the N_2_O meridional dipole is globally reduced compared to ACE‐FTS, with weaker and not significant negative N_2_O trends over the NH. However, WACCM‐REFD1 performs better than WACCM‐REFC1 over the SH, with stronger and significant positive N_2_O trends that reach 30 hPa, similarly to those obtained with ACE‐FTS in the same region. This improvement is possibly related to the changes in the parametrization of the gravity waves (i.e., small‐scale tropospheric waves that drive the BDC) in WACCM version 6 compared to version 4 that followed the increase of its horizontal resolution (Gettelman et al., 2019). This new parametrization results in a good agreement between the gravity waves simulated by WACCM and the observations in the Tropics (Alexander et al., 2021). For tracers, the favorable effect of adjusting the parameterization of the gravity waves in WACCM was shown for ozone in the extratropical SH by Mills et al. (2017). Over the same region, the improved N_2_O trends in WACCM‐REFD1 compared to WACCM‐REFC1 could be attributed to the new parameterization of the gravity waves. This beneficial impact would be consistent with the results of M2020, which showed similar improvements in the N_2_O climatologies between two WACCM versions differing by the parametrization of gravity waves over the SH.

In the lowermost stratosphere (pressure greater than 100 hPa), all models and ACE‐FTS show positive N_2_O trends, resulting from the constant increase in the troposphere. However, the N_2_O increase in the lowermost stratosphere (below 70 hPa) over the Tropics and the NH is not significant in ACE‐FTS, contrary to the model simulations. This difference could be related to the stronger trends in the tropopause rise in the models: around 50 m/decade in CCMs (including WACCM) and ERA5 (Darrag et al., 2022; Pisoft et al., 2021) compared to the observations (around 35 m/decade, Darrag et al., 2022) when using the tropopause definition from the World Meteorological Organization.

### 4.2 Trends in the Model Space

**Figure 4:**
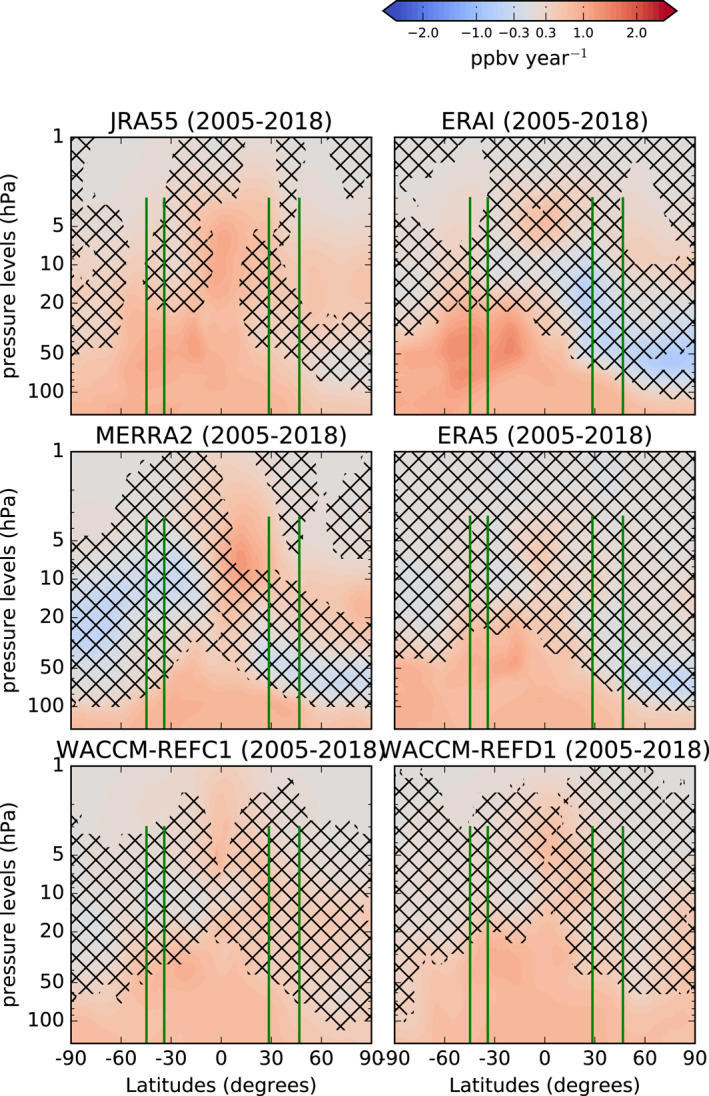
As in Figure 3, but in the model space.

Figure 4 shows the N_2_O trends as in Figure 3, but without applying the ACE‐FTS spatial and temporal sampling. A comparison between each model simulation in the observation and model space (respectively, Figures 3 and 4) reveals large differences in the N_2_O decadal trends. Generally, the sampling of the ACE‐FTS observations enhances the trends simulated by the models, both in the negative and positive directions. For the ERA5 simulation, the significantly negative trend in the NH in observational space becomes insignificant in model space. In addition, one notes immediately that the N_2_O trends in the WACCM simulations change sign, with negative trends in the NH in the observational space becoming weakly positive in model space. In particular for WACCM‐REFD1, the N_2_O trends over the northern mid‐latitudes in the mid‐low stratosphere substantially increase from −0.5 ppbv year^−1^ in observational space to 0.3 ppbv year^−1^ in native model space. However, this difference is not significant because neither of the N_2_O trends above that region is statistically significant with 95% probability.

For satellite measurements, the impact of the sampling in the detection of trends in long‐lived species (including N_2_O) has been evaluated in Millán et al. (2016). They concluded that large errors may arise in the detected trends for coarse and non‐uniform sampling obtained with occultation instruments (such as ACE‐FTS), and that long time scales are required for a robust trend detection from these datasets. Such errors also occur in the models when they are sampled in space and time as the observations. In particular, within the DLM, the non‐uniform time sampling of ACE‐FTS considerably increases the standard deviation of the error in the N_2_O time series, which is zero for regular time sampling. This difference plays a role when deriving trends over these relatively short (decadal) time scales. For example, the non‐uniform ACE‐FTS sampling applied to the ERA5 output results in negative N_2_O trends that are four times stronger compared to the native grid above the northern mid‐latitudes between 50 and 70 hPa. For WACCM, the issue of downsampling was also raised by Garcia et al. (2011) when comparing mean AoA trends obtained from balloon‐borne observations and simulated by the model. Garcia et al. (2011) showed that sampling the model as the observations would deliver positive and non‐significant mean AoA trends, similarly to the observations. We find consistent results with the WACCM simulations: sampling the WACCM output as the observations drives the N_2_O trends toward the observed values. In addition, the non‐significant negative N_2_O trends simulated by WACCM are compatible with the non‐significant positive mean AoA trends found by Garcia et al. (2011) when downsampling WACCM at the mean AoA observations. Hence, the ACE‐FTS sampling exaggerates the simulated N_2_O trends obtained with the DLM in the stratosphere.

In order to understand the trends from the models and compare them with other modeling studies, we now focus on the N_2_O trends obtained from the model datasets in model space (Figure 4). We mentioned earlier that the mean AoA and the N_2_O abundances are inversely correlated in the Tropics and above the lower stratospheric mid‐latitudes. Thus, the stratospheric N_2_O trends have opposite signs compared to trends of mean AoA. For ERAI, the meridional N_2_O trend dipole is consistent with mean AoA trends derived over a shorter period with the same CTM (Chabrillat et al., 2018) and also with different CTMs (Han et al., 2019; Ploeger et al., 2015; Ploeger & Garny, 2022). ERAI shows positive N_2_O trends in the equatorial upper stratosphere, around 5 hPa, which is consistent with the findings of Galytska et al. (2019) using the same reanalysis to drive a different CTM in that region, but no significant trend can be found with ERA5 in the upper stratosphere. The ERA5 simulation confirms the meridional dipole in the mid‐lower stratosphere of ERAI, although the negative N_2_O trend over the NH is not statistically significant at 95% probability. The results obtained with ERA5 are consistent with recent N_2_O and mean AoA trends obtained with a different CTM over a very similar period (Ploeger & Garny, 2022).

Above the southern mid‐latitudes in the mid‐lower stratosphere, the N_2_O trends obtained with MERRA2 are biased low compared to the other models, and do not replicate the hemispheric asymmetry that is visible in the ECMWF reanalyses. Wargan et al. (2018) have shown that the tropopause height has changed in MERRA2 in the past decades, with a decrease in the extratropics and an increase above the Tropics. The pattern of the N_2_O trends in MERRA2 is qualitatively consistent with the changing tropopause height: a rise of the tropopause would lead to positive N_2_O trends, while a sinking tropopause would lead to negative N_2_O trends. In addition, the patterns of the N_2_O trends obtained with MERRA2 disagree with those obtained with the same reanalysis using a different CTM (Ploeger & Garny, 2022), and do not match the mean AoA trends obtained with the same CTM (Chabrillat et al., 2018), at least in the regions where the inverse relationship between N_2_O and mean AoA holds (Galytska et al., 2019). The large differences between JRA55 and MERRA2 and the ECMWF reanalyses in the extratropical mid‐stratosphere highlight that decadal changes in the stratospheric transport are not as robustly detected in JRA55 and MERRA2 as in the ERAI and ERA5. In the equatorial lower stratosphere, all the CTM simulations deliver a significant N_2_O increase, which can partly be attributed to the effect of the tropopause rise that was robustly detected in the reanalyses in the past decades (Manney & Hegglin, 2018).

WACCM does not simulate the N_2_O decrease in the northern polar stratosphere seen in the ECMWF reanalyses, but rather a global N_2_O increase that is largest in the lower stratosphere. The N_2_O increase in the tropical lower stratosphere can be related to the structural changes of the stratosphere in response to global warming that were robustly predicted in CCMs (e.g., Eichinger & Šácha, 2020; Oberländer‐Hayn et al., 2016; Šácha et al., 2019). As discussed in the previous Section, WACCM‐REFD1 improves the representation of the N_2_O trends with respect to WACCM‐REFC1 in the southern mid‐latitudes. The newer WACCM version simulates a significant N_2_O increase up to 20 hPa, which makes the N_2_O trends in the southern mid‐lower stratosphere more similar to those from ERAI and ERA5, even though the decreasing N_2_O trends in the NH are not reproduced. As discussed in the previous Section, this improvement in the N_2_O trends over the SH could be attributed to the adjusted parametrization of gravity waves in version six of WACCM compared to its version 4.

**Figure 5:**
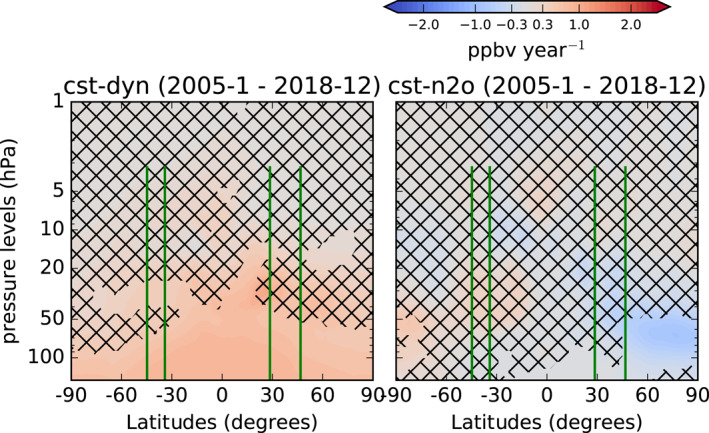
Latitude‐pressure cross sections of N_2_O linear trends (pptv year^−1^) obtained from the DLM from a BASCOE run driven by ERA5 with fixed dynamics and increasing N_2_O (left panel), and from the same model setup but with N_2_O kept constant at the surface and time‐varying dynamics (right panel). The black crosses indicate grid‐points where the probability of positive/negative N_2_O changes is smaller than 95%. The green vertical lines identify the position of the FTIR stations together with their vertical coverage.

Figure 5 shows the N_2_O trends obtained from the two sensitivity tests done with ERA5 (cst‐dyn and cst‐N_2_O). As expected, the cst‐dyn experiment does not simulate any N_2_O decrease in the stratosphere, showing only a steady N_2_O increase as a consequence of the constant buildup at the surface. Between 30 and 50 hPa, the N_2_O increase in the SH is significant with 95% probability, while this is not the case over the NH. This difference can be attributed to the larger variability of the NH over one QBO cycle compared to the SH due to its larger wave activity (Scaife & James, 2000), and was already shown for the significance of ozone trends (Shepherd, 2008). This highlights the importance of considering a sufficiently long period for the trend detection in the stratosphere (Garcia et al., 2011; Hardiman et al., 2017; Strahan et al., 2020). In particular, the 14 years considered here are sufficient to propagate the N_2_O increase to the mid‐stratospheric mid‐latitudes in the SH but not in the NH. The cst‐N_2_O sensitivity test confirms that the extratropical N_2_O trends in the mid‐lower stratosphere are due to the impact of changes in the stratospheric transport. In addition, Ploeger and Garny (2022) recently showed that structural changes of the stratospheric circulation also determine the hemispheric asymmetry in the N_2_O trends in the reanalyses. Contrarily to the cst‐dyn experiment, a changing dynamics impacts the sign of the obtained trends, with an N_2_O decrease above the NH and increase in the SH. From these sensitivity tests, we show that both the mean stratospheric transport and its decadal changes can contribute to the hemispheric asymmetry in the recovered N_2_O trends. The mean stratospheric transport contributes with differences in the significance of the N_2_O trends, and the decadal changes in the stratospheric transport with differences in their respective signs.

## 5 N_2_O TEM Budget

This section further investigates the N_2_O trends from the model simulations using the TEM budget. Equation 2 allows separating the contributions of the residual circulation and mixing terms (respectively, *ADV* and *MIX*) to the N_2_O rate of change. In particular, we aim to identify the contributions from changes in the *ADV* and *MIX* terms to the N_2_O trends shown in the previous section (Figure 4). To that end, we compute the changes of the *ADV* and *MIX* terms as the differences between their linear fits (i.e., the *μ*_*t*_ term of Equation 3) at the end and the beginning of the considered period. A similar analysis was also done in the recent study of Abalos et al. (2020), who used the outputs of several CCMs to compute changes of the TEM budget terms of synthetic tracers. For a detailed description of the climatologies of the *ADV* and *MIX* terms, we refer to M2020.

**Figure 6:**
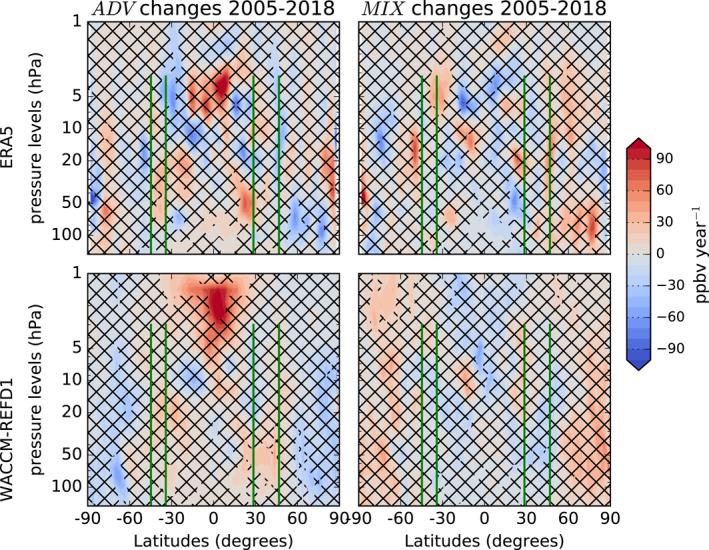
Latitude‐pressure cross sections of the changes of the advection term (A_*z*_ + A_*y*_, left panels, ppbv year^−1^) and mixing term (M_*z*_ + M_*y*_, right panels, ppbv year^−1^) of the TEM N_2_O budget for ERA5 (top) and WACCM‐REFD1 (bottom) for 2005–2018. The black crosses indicate grid‐points where the probability of positive/negative N_2_O changes is smaller than 95%. The green vertical lines identify the position of the FTIR stations together with their vertical coverage.

Here, we provide a qualitative analysis of the contributions from changes in the advection and mixing to the N_2_O trends, by comparing the signs of the changes of *ADV* and *MIX* with those of the N_2_O trends discussed in the previous section. With this approach, we aim to evaluate separately the contributions from changes in *ADV* and *MIX* to the N_2_O trends, and we do not consider their compensation mechanisms that arise from the common driving from the breaking of tropospheric waves (M2020). The complete N_2_O TEM budget also includes the chemistry term $\bar{S}$ (i.e., loss due to photolysis, Tian et al., 2020), which is large in the tropical mid‐high stratosphere, and the residual term $\bar{\epsilon }$, which accounts for all the processes not resolved by the TEM analysis (including mixing on unresolved scales, Equation 2). Figure 6 shows the latitude‐vertical cross sections of those changes in the *ADV* and *MIX* terms over 2005–2018. We limit the analysis to the simulations by ERA5 and WACCM‐REFD1 in order to investigate further the differences in their N_2_O trends discussed in the previous section. In the following, we refer to N_2_O trends discussed in the previous section as “direct” N_2_O trends, in order to distinguish them from the N_2_O changes derived from changes in the *ADV* and *MIX* contributions.

Considering the changes in the *ADV* term, the ERA5 and WACCM‐REFD1 simulations agree over the tropical high stratosphere (above 5 hPa), with a significant increase of the N_2_O abundances due to the impact of the residual circulation, similar to the weak positive direct N_2_O trends in the same region in both simulations. Over this region, the main contribution to the *ADV* term is the transport due to the vertical component of the residual circulation, that is, $-w^{*}{\bar{\chi }}_{z}$ in Equation 1 (M2020). We found that the *ADV* changes are mainly due to an enhanced tropical upwelling − w* over the tropical high stratosphere rather than to changes of the N_2_O vertical gradients ${\bar{\chi }}_{z}$ (not shown). These results of *ADV* changes agree with recent studies showing a strengthening of the advective part of the BDC over the Tropics over longer periods in ERA5 (Diallo et al., 2021) and in version 6 of WACCM (Abalos et al., 2021). In the northern subtropics in the lower stratosphere, both WACCM‐REFD1 and the CTM driven by ERA5 consistently simulate positive N_2_O changes due to an enhanced *ADV* contribution that can contribute to the positive direct N_2_O trend over the same region. This significant contribution from *ADV* can be associated with the strengthening of the shallow branch of the BDC (Lin & Fu, 2013), which was recently detected in ERA5 using the AoA diagnostic (Ploeger et al., 2021) and predicted by CCMs (Butchart, 2014). In the mid‐latitudes in both hemispheres, the changes in the *ADV* term are largely non‐significant in both datasets, especially in WACCM‐REFD1, and do not correspond to any direct N_2_O trend, preventing to draw robust conclusions. In the polar regions, WACCM‐REFD1 and the CTM driven by ERA5 disagree over the Antarctic, where the CCM simulates negative *ADV* changes, while the CTM shows positive non‐significant changes across almost the whole stratosphere. In the Arctic, the two simulations agree well in the lower stratosphere (below 30 hPa) with negative changes of the *ADV* term that can explain the negative direct N_2_O trend and are consistent with the enhanced *ADV* term over the tropical region.

Concerning the *MIX* term, its changes are more irregular compared to those of the *ADV* term in both WACCM‐REFD1 and ERA5, and do not correspond to the direct N_2_O trends over the NH. In addition, the *MIX* changes obtained with the CCM largely disagree with those from the CTM and their significance is considerably smaller. Large differences between WACCM and the CTM driven by reanalyses were already shown by M2020 for the climatologies of the mixing terms of the N_2_O TEM budget. Furthermore, the weaker effect of mixing in WACCM‐REFD1 compared to ERA5 is consistent with Dietmüller et al. (2017), who found weaker AoA trends in the resolved aging by mixing in a free‐running CCM compared to its specified‐dynamics version and a reanalysis. Concerning the *MIX* term in ERA5, in the SH between 10 and 30 hPa, there is enhanced poleward mixing that transports N_2_O from the subtropics (where *MIX* changes are negative) to the mid‐latitudes (where the *MIX* changes are positive). Such positive N_2_O changes in the southern mid‐latitudes can be associated with the positive but not significant direct N_2_O trends over the same region. The role of both resolved and unresolved mixing in the decadal BDC trends has been studied in ECMWF reanalyses, especially using AoA (e.g., Dietmüller et al., 2017; Ploeger et al., 2015). Recent studies have associated the N_2_O trend dipole discussed in the previous section with a southward shift of the circulation pattern, which in turn is related to the impact of mixing on the BDC changes (Ploeger et al., 2019; Stiller et al., 2017). Our results with N_2_O from ERA5 confirm the role of mixing processes above the southern mid‐latitudes in determining changes in the N_2_O abundances, and indirectly support the hypothesis of the southward shift of the circulation as a contribution to the dipole structure.

This qualitative analysis suggests that, within the N_2_O TEM framework, changes in the residual circulation have a stronger impact on the direct N_2_O trends compared to those in mixing. However, the ERA5 simulation also delivers significant N_2_O changes due to mixing that are consistent with previous studies using ECMWF reanalyses and that can be associated to the hemispheric asymmetry in the direct N_2_O trends simulated by ERA5.

## 6 Summary and Conclusions

We have evaluated the stratospheric N_2_O columns (12–40 km) and their decadal (2005–2018) rates of change in two versions of WACCM: WACCM‐REFC1 (version 4) and WACCM‐REFD1 (version 6). We compared those changes with ground‐based observations at four ground‐based FTIR stations: Lauder (45°S), Wollongong (34°S), Izaña (28°N) and Jungfraujoch (46°N), with space‐borne measurements from ACE‐FTS, and with the output of the BASCOE CTM driven by four modern reanalyses: ERAI, ERA5, JRA55 and MERRA2. We also studied the latitudinal and vertical distributions of these trends of the N_2_O mixing ratios from model output and satellite measurements, both in the observation and model space. Also, we use the Transformed Eulerian Mean (TEM) budget to investigate further the decadal trends in the BASCOE CTM driven by ERA5 and in WACCM‐REFD1.

The comparison of the stratospheric N_2_O columns reveals a good agreement above Wollongong, and Lauder to a lesser extent, and larger differences above Jungfraujoch and Izaña. The trends in the stratospheric N_2_O columns obtained from FTIR are larger in the SH compared to the NH, which is consistent with hemispherical differences in trends of stratospheric tracers measured at FTIR stations over the past decade (Prignon et al., 2021; Strahan et al., 2020). This hemispheric asymmetry is present in the ECMWF reanalyses but is weaker in WACCM. We find that the decadal trends in the stratospheric N_2_O columns are consistently positive in all cases except for ACE‐FTS observations above Jungfraujoch. However, the vertical resolution of the FTIR retrievals above the NH for N_2_O limits our analysis to one stratospheric column, hence (in this analysis) the detection of potentially negative N_2_O trends in the mid‐stratosphere is hindered by the large N_2_O increase in the lowermost stratosphere which arises from its continuous increase at the surface.

Global and vertically resolved trends of N_2_O volume mixing ratios provide a more detailed picture compared to the stratospheric N_2_O columns obtained from FTIR measurements. The ACE‐FTS measurements show a meridional dipole in the N_2_O trends in the mid‐lower stratosphere, with negative values in the NH mid‐latitudes and positive values in the SH. When applying the temporal and spatial sampling of ACE‐FTS on model datasets, ERAI and ERA5 compare best with the satellite measurements while the other reanalyses and WACCM do not reproduce the meridional dipole in the mid‐lower stratosphere as clearly as the ECMWF reanalyses. However, this application of the irregular sampling of ACE‐FTS to the model output consistently enhances the N_2_O trends, both positive and negative. Using continuous time sampling on native model grids, ERAI, and ERA5 to a lesser extent, still simulate the meridional dipole in the N_2_O trends, consistently with a large number of modeling studies using both idealized and real tracers (e.g., Chabrillat et al., 2018; Ploeger & Garny, 2022; Prignon et al., 2021), but MERRA2, JRA55 and both WACCM versions fail to reproduce the meridional dipole. The inherently limited spatial and temporal sampling of ACE‐FTS, and its effect on N_2_O trends, highlight the necessity to carry out the N_2_O trend analysis discussed here using a satellite instrument with a more regular coverage. To this end, MLS is a very good candidate, once its drift in the N_2_O retrievals will be corrected.

Concerning the WACCM simulations, the too weak hemispheric asymmetries in the trends of the stratospheric N_2_O columns and volume mixing ratios highlights small inter‐hemispheric differences in the stratospheric transport. However, the adjusted parametrization of gravity waves in version 6 of WACCM improves the trends in N_2_O volume mixing ratios above the southern mid‐latitudes compared to its version 4. This highlights the importance of the parametrization of the gravity waves for a correct reproduction of trends in long‐lived tracers in the SH in WACCM. Therefore, we emphasize that modeling groups should continue their efforts in improving the horizontal resolution and adjusting the gravity waves parametrization in CCMs.

We carried out two sensitivity tests using the BASCOE CTM driven by ERA5: one keeping N_2_O constant at the surface with time‐dependent dynamics (cst‐N_2_O), and the other using fixed dynamics with increasing N_2_O at the surface (cst‐dyn). The cst‐N_2_O experiment confirms that the extratropical N_2_O trends in the mid‐lower stratosphere are due to the impact of changes in the stratospheric transport. As expected, the cst‐dyn simulation shows that N_2_O increases everywhere, but the trend over 2005–2018 is not significantly positive in the NH mid‐stratosphere. From these sensitivity tests with ERA5, we confirm that the hemispheric asymmetry of the decadal N_2_O trends arises from decadal changes in the transport strength in combination with structural changes of the transport pattern. For the 2005–2018 period and in the 20–50 hPa layer, the hemispheric asymmetry in the significance of these trends arises from the larger dynamical variability which is found in the northern extratropics on shorter timescales, that is, within one QBO cycle.

We found a strong impact of transport on the stratospheric trends of N_2_O volume mixing ratios for the ERA5 simulations and large differences between ERA5 and WACCM‐REFD1. This prompted us to study the TEM budget of N_2_O in these two datasets, in order to separate the possible impacts of the residual circulation and mixing. For both datasets, the analysis of the TEM budget reveals positive N_2_O changes in the tropical mid‐high stratosphere and negative changes in the northern extratropical lower stratosphere, as a result of enhanced tropical upwelling and extratropical downwelling, respectively. This is in agreement with the acceleration of the advective part of the BDC over this relatively short period both in models (Butchart, 2014) and reanalyses (Ploeger et al., 2019). For the ERA5 simulation, the positive N_2_O trend above the southern mid‐latitudes (part of the meridional dipole) can be due to the impact of changes in resolved mixing, which is consistent with previous studies, both using Age of Air (AoA, Ploeger et al., 2015) and N_2_O (Stiller et al., 2017).

Using a measurable tracer for stratospheric transport studies allows direct comparisons with observations. The rate of change of N_2_O at the surface is well‐known and approximately linear and the chemical losses are limited to the higher stratosphere. In theory, this relatively simple chemistry, combined with its long life, makes N_2_O a very good tracer for stratospheric transport studies. Unfortunately, no ideal observational dataset currently exists for N_2_O‐based investigations such as the present study: FTIR observations generally lack adequate vertical resolution, the N_2_O product from the latest MLS version suffers from an unrealistic drift, and ACE‐FTS has poor spatial and temporal sampling. Here, we showed how model studies of N_2_O trends still provide new insights about the BDC and its changes thanks to properly taking into account the ACE‐FTS sampling, complementary sensitivity tests, and the TEM analysis. In particular, the improvements in version 6 of WACCM compared to version 4 highlight that the next‐generation CCMs can potentially reach the quality of the reanalyses in terms of decadal changes of long‐lived tracers in the stratosphere. Despite the shortcomings of the TEM approach, that is, the difficulty of closing its budget, its combination with sensitivity tests provides new insights on transport changes and their impacts on the composition of the stratosphere. This approach could be extended to other tracers that are both measured and modeled—for example, carbon monoxide, methane, and inorganic fluorine.

## Data Availability

The WACCM and BASCOE CTM data used for the N_2_O trends and TEM comparisons in the study are available at the BIRA‐IASB repository (http://repository.aeronomie.be) via https://dx.doi.org/10.18758/71021071 with CC BY license (Minganti et al., 2022). FTIR data at the various stations are available at https://www-air.larc.nasa.gov/pub/NDACC/PUBLIC/stations/. ACE‐FTS data are available at https://databace.scisat.ca/level2/ace_v4.1/display_data.php. ERA5 data are available at https://cds.climate.copernicus.eu/. ERA‐Interim data are available at https://apps.ecmwf.int/datasets/. JRA‐55 data are available at https://rda.ucar.edu/. MERRA2 data are available at https://disc.gsfc.nasa.gov/datasets/. The DLM source code is available at https://doi.org/10.5281/zenodo.2660704.
